# Engineering Promiscuous Alcohol Dehydrogenase Activity of a Reductive Aminase *Asp*RedAm for Selective Reduction of Biobased Furans

**DOI:** 10.3389/fchem.2021.610091

**Published:** 2021-05-13

**Authors:** Hao-Yu Jia, Zi-Yue Yang, Qi Chen, Min-Hua Zong, Ning Li

**Affiliations:** ^1^ School of Food Science and Engineering, South China University of Technology, Guangzhou, China; ^2^ State Key Laboratory of Bioreactor Engineering, Shanghai Collaborative Innovation Center for Biomanufacturing, East China University of Science and Technology, Shanghai, China

**Keywords:** alcohol dehydrogenases, biobased furans, catalytic promiscuity, protein engineering, reductive aminases

## Abstract

Catalytic promiscuity is a promising starting point for improving the existing enzymes and even creating novel enzymes. In this work, site-directed mutagenesis was performed to improve promiscuous alcohol dehydrogenase activity of reductive aminase from *Aspergillus oryzae* (*Asp*RedAm). *Asp*RedAm showed the cofactor preference toward NADPH in reductive aminations, while it favored NADH in the reduction reactions. Some key amino acid residues such as N93, I118, M119, and D169 were identified for mutagenesis by molecular docking. Variant N93A showed the optimal pH and temperature of 8 and 30°C, respectively, in the reduction of 5-hydroxymethylfurfural (HMF). The thermostability was enhanced upon mutation of N93 to alanine. The catalytic efficiency of variant N93A (*k*_cat_/*K*_m_, 23.6 mM^−1^ s^−1^) was approximately 2-fold higher compared to that of the wild-type (WT) enzyme (13.1 mM^−1^ s^−1^). The improved catalytic efficiency of this variant may be attributed to the reduced steric hindrance that stems from the smaller side chain of alanine in the substrate-binding pocket. Both the WT enzyme and variant N93A had broad substrate specificity. *Escherichia coli* (*E. coli*) cells harboring plain vector enabled selective reduction of biobased furans to target alcohols, with the conversions of 35–95% and the selectivities of >93%. The introduction of variant N93A to *E. coli* resulted in improved substrate conversions (>98%) and selectivities (>99%).

## 1 Introduction

Enzymes are well known for their exquisite chemo-, regio-, and stereoselectivities. In addition to the reactions that they have evolved for, nonetheless, more enzymes have been found to be capable of promiscuously catalyzing mechanistically distinct transformations (termed catalytic promiscuity) (Kazlauskas, 2005). Enzyme catalytic promiscuity presumably plays a crucial role in biological evolution (Khersonsky et al., 2006). From the synthetic chemistry point of view, the promiscuous yet low activities may be an attractive starting point for improving the existing enzymes and even creating enzymes for novel synthetic routes that are currently not available (Bornscheuer and Kazlauskas, 2004; Brandenberg et al., 2017; Leveson-Gower et al., 2019).

Reductive aminases (RedAms), a subclass of imine reductases (IREDs), have evolved to be capable of catalytic formation of imines as well as their reduction, namely, reductive amination reactions (Aleku et al., 2017; Sharma et al., 2017; Ducrot et al., 2020). We recently found that a RedAm from *Aspergillus oryzae* (*Asp*RedAm) (Aleku et al., 2017) possessed promiscuous alcohol dehydrogenase (ADH) activity, which enabled 5-hydroxymethylfurfural (HMF) to be selectively but slowly reduced to 2,5-bis(hydroxymethyl)furan (BHMF). Likewise, Lenz et al. reported that IREDs could promiscuously reduce an activated ketone (Lenz et al., 2017). Also, the primary function of IREDs was identified as a promiscuous activity of DHs such as β-hydroxyacid dehydrogenases and glucose dehydrogenase (Roth et al., 2017; Lenz et al., 2018). More importantly, a variety of engineering strategies were successfully applied to turn the promiscuous activities of many enzymes into the primary ones for organic synthesis (Svedendahl et al., 2005; Leveson-Gower et al., 2019). Lenz et al. described the generation of new IREDs from β-hydroxyacid dehydrogenases by single amino acid substitutions (Lenz et al., 2018). Amine dehydrogenases (AmDHs), a type of useful enzymes catalyzing the conversion of ketones to enantiomerically pure amines, were created from amino acid dehydrogenases (Tseliou et al., 2019; Liu et al., 2020). Recently, Tseliou reported that l-lysine-ε-dehydrogenase variants showing dual AmDH/ADH activities were applied for direct conversion of alcohols to amines using a single enzyme (Tseliou et al., 2020).

The concerns on fossil resource crisis and environmental issues (e.g., global warming) motivate the production of chemicals and fuels from renewable, abundant, and carbon-neutral biomass (Tuck et al., 2012; Sheldon, 2014; Sheldon, 2018). Biobased furans obtained via carbohydrate dehydration are versatile platform chemicals bridging the gap between biomass and biobased chemicals as well as between biomass and biofuels (van Putten et al., 2013; Mariscal et al., 2016; Galkin and Ananikov, 2019; Shen et al., 2020). Due to their high reactivity, these biobased furans could be readily reduced to value-added furan alcohols such as BHMF and furfuryl alcohol (Li et al., 2016; He et al., 2018; Hu et al., 2018; Petri et al., 2018; Yan et al., 2019; Zhang et al., 2019; Amarasekara et al., 2020), important building blocks in polymer, food, and pharmaceutical industries. Recently, our group has focused our attention on biocatalytic valorization of biobased furans (Qin et al., 2015; Li et al., 2017; Zhang et al., 2017; Jia et al., 2019b; Zhang et al., 2020a; Wang et al., 2020; Wen et al., 2020). To continue our interest in the furan upgrading, the reduction of HMF was used as the model reaction for evaluating the ADH activities of the engineered enzymes. In this work, a structure-guided semirational strategy was used to improve the promiscuous ADH activity of *Asp*RedAm. Some key amino acid residues in the active site were identified as the potential hotpots for molecular modifications of *Asp*RedAm. Like l-lysine-ε-dehydrogenase variants mentioned above (Tseliou et al., 2020), on the other hand, the engineered *Asp*RedAms may be potential biocatalysts capable of direct single-enzyme catalytic *N*-alkylation of amines using alcohols, due to its dual RedAm/ADH activities. A comparison study on the enzyme properties of the wild-type (WT) enzyme and variant N93A was conducted, together with the evaluation of their substrate spectra. *Escherichia coli* (*E. coli*) whole cells harboring variant N93A were used for the synthesis of biobased furan alcohols from furans.

## 2 Materials and Methods

### Chemical and Biological Materials

HMF **1** (98%) and cyclohexanone (99%) were purchased from Aladdin (Shanghai, China). Furfural **2** (99%), BHMF (98%), and propylamine (98%) were obtained from Macklin Biochemical Co., Ltd. (Shanghai, China). 5-Methoxymethylfurfural **3** (97%) was bought from Adamas Reagent Ltd. (Shanghai, China). 5-Methylfurfural **4** (97%) and furfuryl alcohol (98%) were purchased from TCI (Japan). NAD(P)H was obtained from Yuanye Biotech (Shanghai, China). 5-Methylfurfuryl alcohol (98%) was purchased from Apollo Scientific Ltd. (United Kingdom). 5-Methoxymethylfurfuryl alcohol was synthesized and purified according to a recent method (Cheng et al., 2020). Other chemicals are of analytical grade and commercially available.

*Escherichia coli* BL21(DE3) and plasmid pET-28a were obtained from Novagen Inc. (Madison, United States). Protein marker, restriction endonucleases *Xho*I and *Nde*I, and T4 DNA ligase were purchased from Thermo Fisher Scientific GmbH (Schwerte, Germany). Q5 High-Fidelity DNA polymerase was obtained from New England Biolabs Inc. (United States). *Dpn*I endonuclease was purchased from TaKaRa Biotechnology Co., Ltd. (Dalian, China). Isopropyl β-d-1-thiogalactopyranoside (IPTG), DNA marker, and ampicillin were purchased from Sangon Biotech (Shanghai, China). Glucose dehydrogenase (GDH) from *Bacillus megaterium* (20 U/mg) was prepared according to a previous method (Jia et al., 2019a). Cloning, heterologous expression, site-directed mutagenesis, and protein purification of *Asp*RedAm and variants were available as Supplementary Material.

### Enzyme Assay

The ADH activities were determined spectrophotometrically by measuring the decrease in the NAD(P)H absorbance at 340 nm under pH 8/9 and 30°C. The reaction mixtures contained 43 μg/ml *Asp*RedAm/37 μg/ml N93A, 5 mM HMF, and 0.2 mM NAD(P)H in Tris–HCl buffer (100 mM, pH 8 for N93A; pH 9 for *Asp*RedAm). One unit (U) was defined as the amount of enzyme that catalyzes the oxidation of 1 μmol of NAD(P)H per minute under the above reaction conditions. The protein concentration was measured with Bradford method (Bradford, 1976), using bovine serum albumin as the standard.

### General Procedure for Biocatalytic Reduction of Furans

Typically, 2 ml of Tris–HCl buffer (100 mM, pH 8) containing 25 mM of furans, 30 mM glucose, and 50 mg/ml of whole cells (wet weight) was incubated at 30°C and 150 rpm. Aliquots were withdrawn from the reaction mixtures at specified time intervals and diluted with the corresponding mobile phase prior to HPLC analysis. The conversion was defined as the ratio of the consumed substrate amount to the initial substrate amount (in mol). The selectivity was defined as the ratio of the amount of the desired product to the sum of all the products (in mol). All the experiments were conducted at least in duplicate, and the values were expressed as the means ± standard deviations.

### Molecular Docking

Molecular docking was performed for both the WT and variant N93A in complex with substrate HMF and NADH. Modeller (version 9.13) was used to generate the 3D structure of variant N93A with NADH using the WT enzyme (PDB ID: 5G6R) as the template. Then AutoDock (version 4.2) was used for substrate docking, and 250 docking poses were obtained for each system. The simulated systems were named WT-NADH-HMF and N93A-NADH-HMF, respectively. Configuration clusters were analyzed for the reasonable binding mode with the highest docking energy.

### HPLC Analysis

The reaction mixtures were analyzed on a Zorbax Eclipse XDB-C18 column (4.6 mm × 250 mm, 5 μm, Agilent, United States) by using a reversed phase HPLC (Waters, United States) equipped with a 996 PDA detector. Unless otherwise stated, the mobile phase for HPLC analysis was a mixture of acetonitrile/0.4% (NH_4_)_2_SO_4_ aqueous solution (pH 3.5, 1:9, v/v) at a flow rate of 0.6 ml/min. A mixture of acetonitrile/0.4% (NH_4_)_2_SO_4_ aqueous solution (pH 3.5, 2:8, v/v) at a flow rate of 0.8 ml/min was used for monitoring the transformation of compound **3**, while the mixture of acetonitrile/0.02% H_3_PO_4_ solution (4:6, v/v) acted as the mobile phase at a flow rate of 0.3 ml/min for monitoring the transformation of **4**. HPLC chromatograms are available as Supplementary Material.

## 3 Results and Discussion

### Site-Directed Mutagenesis

**FIGURE 1:**
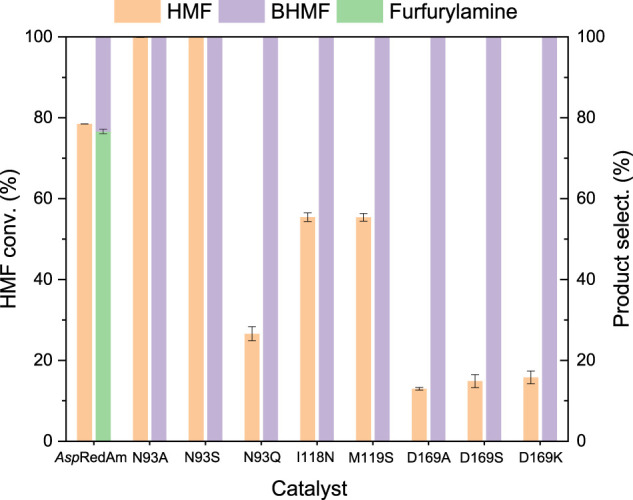
Comparison of the catalytic performances of variants in the conversion of HMF. Reaction conditions: 5 mM HMF, 100 mM propylamine, 30 mM glucose, 0.2 mM NADPH, 0.5 mg/ml (7.8 μM) *Asp*RedAm and its variants, 0.5 mg/ml GDH, 1 ml Tris–HCl buffer (0.1 M, pH 8), 150 rpm, 25°C, 24 h; tuning final pH to 9 after the addition of propylamine. The values were obtained from two independent experiments.

Based on the crystal structure of *Asp*RedAm (Aleku et al., 2017) and the results of this protein docking with HMF and cofactor (Supplementary Figure 1), the residues within 4 Å around substrate HMF such as N93, I118, M119, A120, V121, and D169 were identified as the potential hotspots for mutation, since they may interact with substrate and cofactor. The catalytic performances of variants showing high selectivities toward BHMF were summarized in Figure 1, with the WT enzyme as control. In addition to the reductive amination product (*N*-propyl furfurylamine), the reduction product BHMF (the selectivity of around 23%) was also produced in reductive amination of HMF by *Asp*RedAm, indicating the promiscuous ADH activity of this enzyme. Interestingly, these variants did not show the RedAm activities with HMF as substrate, but ADH activities (Figure 1), since no formation of *N*-propyl furfurylamine was observed. Notably, the RedAm activities of some variants (e.g., N93A) toward cyclohexanone remained (data not shown). Besides N93 and D169 (Aleku et al., 2017), other residues I118 and M119 were also recognized as the new important sites for reductive amination reactions. Among the mutants tested, variants N93A and N93S provided the best results in the reduction of HMF, with the substrate conversions of >99% and the BHMF selectivities of >99% (Figure 1). The ADH activities of the two variants were further compared in the absence of amine donor (Supplementary Table 2). Interestingly, mutation of N93 to alanine resulted in a 6.6-fold increase in the ADH activity, while no significantly improved activity was found with variant N93S. Like asparagine, serine has a more hydrophilic and larger side chain compared to alanine, which may result in unfavorable interactions with NADPH and/or substrate. Thus, variant N93S also showed a low activity. So, the enzyme properties of variant N93A were explored in the subsequent studies.

### Biochemical Characterization

Both RedAms and ADHs are NAD(P)H-dependent enzymes, and cofactors have usually a great effect on their activities. On the other hand, NADPH is costlier than NADH. Therefore, the cofactor preference of the WT enzyme and variant was examined (Table 1). Unexpectedly, the WT enzyme preferred NADH as cofactor in the reduction reactions regardless of substrates (Table 1). However, it favored NADPH in the reductive amination reactions (Supplementary Table 3), which is consistent with the previous results (Aleku et al., 2017). It suggests that there may exist the mechanistic differences between the reduction and reductive amination reactions. According to Borlinghaus et al. (Borlinghaus and Nestl, 2018), all characterized members of the IRED family showed strict NADPH preference in the reductive amination. Variant N93A showed the NADH preference in the reduction of both HMF and cyclohexanone. Although the improvement in the ADH activities was achieved upon mutation, the activity of variant N93A toward HMF remained unsatisfactory (<0.1 U/mg). And it is much lower than those of some natural ADHs in the HMF reduction (Almeida et al., 2008; Laadan et al., 2008). Therefore, further improvement in its ADH activity by molecular modification is required for its future practical applications.

**TABLE 1:** Effect of cofactors on the ADH activities of *Asp*RedAm and variant N93A.

Enzyme	Enzyme activity (mU/mg)			
	HMF		Cyclohexanone	
	NADH	NADPH	NADH	NADPH
*Asp*RedAm	17.0 ± 3.0	0.7 ± 0.0	28.5 ± 0.8	0.9 ± 0.2
N93A	80.1 ± 5.1	4.4 ± 0.3	90.9 ± 5.3	2.7 ± 0.1

Reaction conditions: 15 mM substrate, 0.2 mM NAD(P)H, 43 μg/ml *Asp*RedAm/37 μg/ml N93A, 0.4 ml Tris–HCl buffer (100 mM, pH 9), 30°C. The values were obtained from two independent experiments.

**FIGURE 2:**
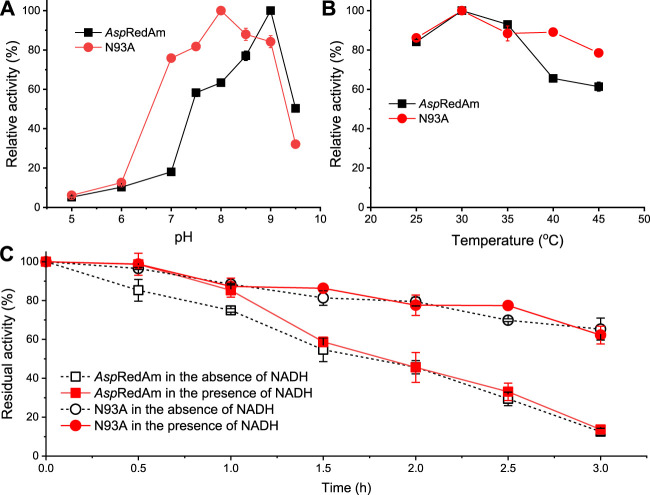
Effect of pH **(A)** and temperature **(B)** on the enzyme activities and thermostability **(C)**. General reaction conditions: 5 mM HMF, 0.2 mM NADH, 43 μg/ml *Asp*RedAm/37 μg/ml N93A, 0.4 ml buffer, 30°C; **(A)** buffer: Na_2_HPO_4_ (100–200 mM) citric acid (50–0 mM) buffer (pH 5–8), Tris–HCl buffer (100 mM, pH 8.5–9), glycine-NaOH buffer (100 mM, pH 9.5); **(B)** 100 mM Tris–HCl buffer (pH 9 for *Asp*RedAm; pH 8 for N93A), 25–45°C; **(C)** after the enzyme was incubated in 100 mM Tris–HCl buffer (pH 9 for *Asp*RedAm; pH 8 for N93A) at 30°C, the enzyme activities were assayed. The values were obtained from two independent experiments.

Effect of pH and temperature on the ADH activities and stability was evaluated (Figure 2). Figure 2A shows that the WT enzyme has the highest activity at pH 9 in the reduction of HMF, which is consistent with that in the reductive amination (Aleku et al., 2017). Its optimal pH slightly decreased to 8 upon mutation of N93 to alanine. Besides, the variant exhibited the relative activities of more than 70% within pH 7–9, indicating its good pH tolerance. As shown in Figure 2B, the optimal temperature of both the WT and variant was 30°C in the reduction, which is in agreement with that in the reductive amination (Aleku et al., 2017). Additionally, variant N93A displayed higher activities than the WT enzyme at 40–45°C (Figure 2B). The thermostability of the WT enzyme and mutant was assessed (Figure 2C). Intriguingly, variant N93A was found to be more thermally stable than the WT enzyme, regardless of whether cofactor is present or absent. In the absence of NADH, for example, variant N93A retained 65% of the residual activity after incubation of 3 h, while only 12% of the residual activity was observed with the WT enzyme. It may be one of the promising features of this variant for its application in synthetic chemistry.

Table 2 shows the effect of metal ions on the ADH activities of *Asp*RedAm and variant N93A. Except for Al^3+^, almost all the metals tested exerted a great inhibitory effect on the ADH activities of the WT enzyme. The precipitation (possibly protein) occurred in the presence of Co^2+^ and Ni^2+^, resulting in no observed activities. A very low activity of *Asp*RedAm was recorded in the presence of Mn^2+^. Likewise, the activities of variant N93A were significantly inhibited by Mn^2+^ and Co^2+^, although no precipitation happened.

**TABLE 2:** Effect of metal ions on the ADH activities of *Asp*RedAm and variant N93A.

Metal	Relative activity (%)	
	*Asp*RedAm	N93A
Control	100	100
Ca^2+^	24 ± 1	46 ± 2
Cu^2+^	69 ± 5	61 ± 2
Mg^2+^	75 ± 6	56 ± 6
Mn^2+^	3 ± 1	11 ± 2
Al^3+^	97 ± 5	73 ± 1
Co^2+^	n.a.	4 ± 1
Ni^2+^	n.a.	58 ± 4

n.a., no activity.

Reaction conditions: 5 mM HMF, 0.2 mM NADH, 43 μg/ml *Asp*RedAm/37 μg/ml N93A, 1 mM metal, 0.4 ml Tris–HCl buffer (100 mM, pH 9 for *Asp*RedAm; pH 8 for N93A), 30°C. The values were obtained from two independent experiments.

**FIGURE 3:**
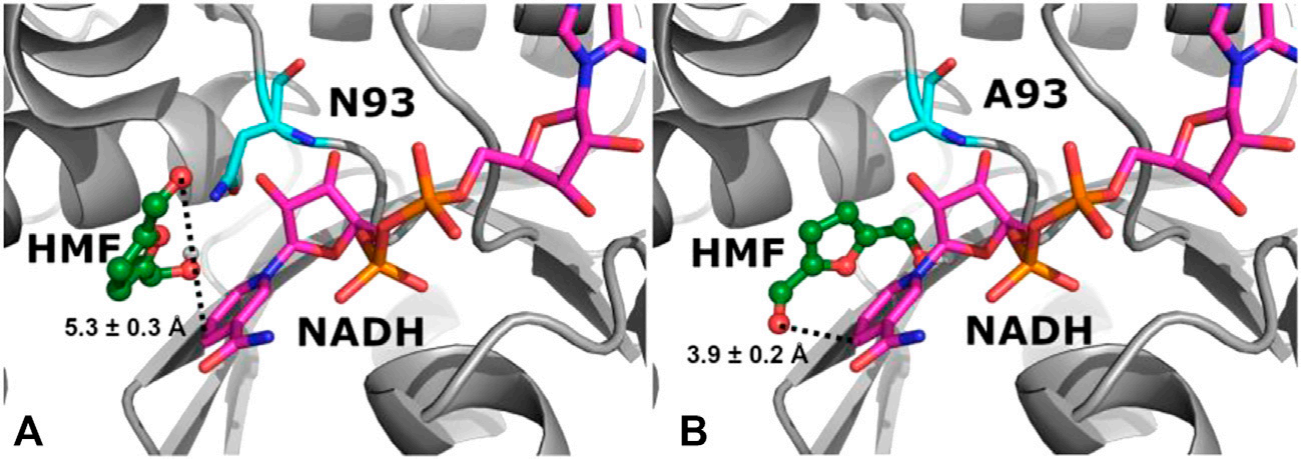
Binding mode analysis of HMF and NADH in WT **(A)** and variant N93A **(B)**. The 3D structures of enzymes in complex with HMF and NADH were constructed from NADPH-containing homology models by removing phosphate in silico by Modeller (version 9.13), and molecular docking was performed by AutoDock (version 4.2).

The reaction kinetics were studied in the HMF reduction catalyzed by *Asp*RedAm and variant N93A, and the kinetic constants were determined based on the Lineweaver–Burk plots (Table 3). It is worth noting that no substrate inhibition toward enzymes is observed within the substrate concentration range tested (data not shown). The *K*_m_ value of the WT enzyme was approximately 1.3 μM toward HMF, while the corresponding value of variant N93A approached 4.1 μM. The increased *K*_m_ value of the mutant indicates its lower substrate affinity toward HMF compared to that of the WT enzyme. Nonetheless, variant N93A provided a greatly improved *k*_cat_ value compared to the WT enzyme (96.7 vs 17.5 ms^−1^). Therefore, the catalytic efficiency of the mutant (*k*_cat_/*K*_m_) was approximately 2-fold higher than that of the WT enzyme. To get mechanistic insights into the increased catalytic activity upon N93A mutation, molecular docking was performed for both the WT enzyme and variant N93A in complex with substrate HMF and NADH (Figure 3). As shown in Figure 3, the distance between C_NADH_ and O_LIGAND_ decreased from 5.3 ± 0.3 Å in WT-NADH-HMF to 3.9 ± 0.2 Å in N93A-NADH-HMF, and the shorter distance is more appropriate for hydride transfer. Besides, the increased docking energy was observed in N93A-NADH-HMF (−4.0 ± 0.1 kcal/mol) compared to that (−3.7 ± 0.2 kcal/mol) in WT-NADH-HMF, indicating stronger binding of substrate to variant N93A. The reason for the above-mentioned results may be that the smaller side chain of Ala93 allows substrate to bind more strongly in the binding pocket, due to its reduced steric hindrance.

**TABLE 3:** Kinetic constants of *Asp*RedAm and variant N93A in the HMF reduction.

Enzyme	*K*_m_ (×10^−3^ mM)	*k*_cat_ (×10^−3^ s^−1^)	*k*_cat_/*K*_m_ (mM^−1^ s^−1^)
*Asp*RedAm	1.3 ± 0.2	17.5 ± 0.3	13.1 ± 1.8
N93A	4.1 ± 0.7	96.7 ± 7.6	23.6 ± 2.0

Reaction conditions: 1 μM-5 mM HMF, 0.2 mM NADH, 43 μg/ml *Asp*RedAm/37 μg/ml N93A, 0.4 ml Tris–HCl buffer (100 mM, pH 9 for *Asp*RedAm; pH 8 for N93A), 30°C. The values were obtained from two independent experiments.

### Substrate Spectra

It is well known that the substrate tolerance of enzymes is of great importance for their synthetic applications. Broad substrate specificity enables enzymes to be facilely applied to produce a variety of chemicals. Therefore, the substrate scopes of the WT and mutant were investigated (Table 4). *Asp*RedAm was reported to have broad substrate specificity in reductive amination (Aleku et al., 2017). Also, the WT enzyme could accept all the tested chemicals as substrates in the reduction reactions, in spite of low promiscuous ADH activities (<35 mU/mg), and compound **11** was considered optimal. Increased activities were observed with variant N93A, and the highest activity of approximately 96 mU/mg was obtained when compound **7** acted as substrate. In general, variant N93A displayed higher activities toward phenyl-containing substrates (compounds **9**–**14**) than furan aldehydes (**1**–**8**). The similar phenomenon was also found in the case of the WT enzyme. Recently, we also found that aldehyde dehydrogenases preferred benzylaldehydes to furan aldehydes as substrates (Zhang et al., 2020b). Therefore, it seems to be a common catalytic feature of enzymes, likely because of the higher richness of benzene-based chemicals in nature. In addition, the mutant as well as the WT enzyme could accept aliphatic ketones (**15** and **16**) as substrates. Like the WT enzyme, variant N93A had broad substrate specificity, indicating its application potential in synthetic chemistry.

**TABLE 4:** Substrate spectra of *Asp*RedAm and variant N93A.

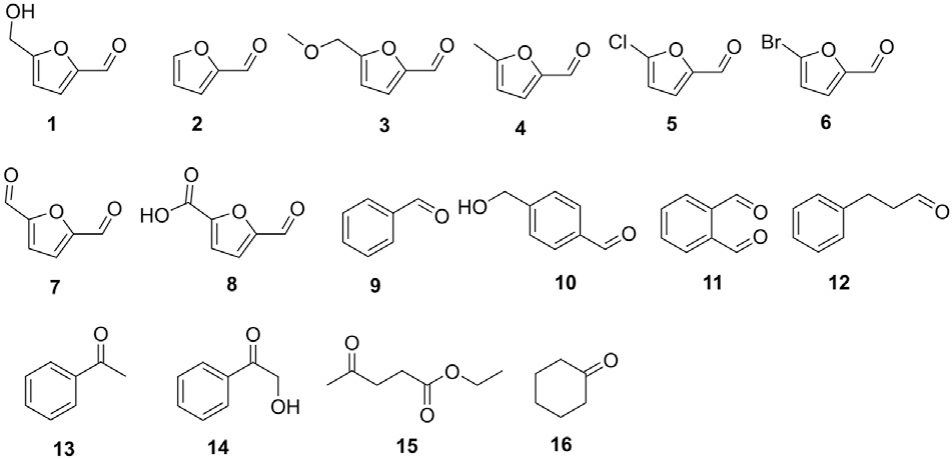		
Substrate	Specific activity (mU/mg)	
	*Asp*RedAm	Variant N93A
**1**	18.9 ± 1.3 (56%)	83.0 ± 0.2 (87%)
**2**	20.9 ± 0.9 (62%)	70.4 ± 6.4 (74%)
**3**	20.5 ± 2.1 (61%)	75.2 ± 2.3 (78%)
**4**	19.5 ± 1.9 (58%)	69.8 ± 1.8 (73%)
**5**	16.9 ± 1.4 (50%)	54.6 ± 5.1 (57%)
**6**	25.1 ± 1.0 (75%)	69.9 ± 1.7 (73%)
**7**	10.3 ± 0.2 (31%)	41.4 ± 4.1 (43%)
**8**	30.9 ± 2.1 (92%)	**95.9 ± 3.4 (100%)**
**9**	29.7 ± 0.8 (89%)	74.9 ± 1.3 (78%)
**10**	25.3 ± 0.0 (75%)	80.1 ± 4.1 (84%)
**11**	**33.6 ± 0.9 (100%)**	82.0 ± 1.8 (86%)
**12**	30.5 ± 0.2 (91%)	75.0 ± 1.1 (78%)
**13**	31.3 ± 1.6 (93%)	80.3 ± 6.6 (84%)
**14**	29.5 ± 1.9 (88%)	83.4 ± 0.3 (87%)
**15**	31.5 ± 0.3 (94%)	91.1 ± 2.5 (95%)
**16**	31.4 ± 1.9 (94%)	82.5 ± 2.1 (86%)

Reaction conditions: 5 mM substrate, 0.2 mM NADH, 43 μg/ml *Asp*RedAm/37 μg/ml N93A, 0.4 ml Tris–HCl buffer (100 mM, pH 9 for *Asp*RedAm; pH 8 for N93A), 30°C. Relative activities were presented in parentheses. The values were obtained from two independent experiments.

### Application for the Reduction of Furans

**FIGURE 4:**
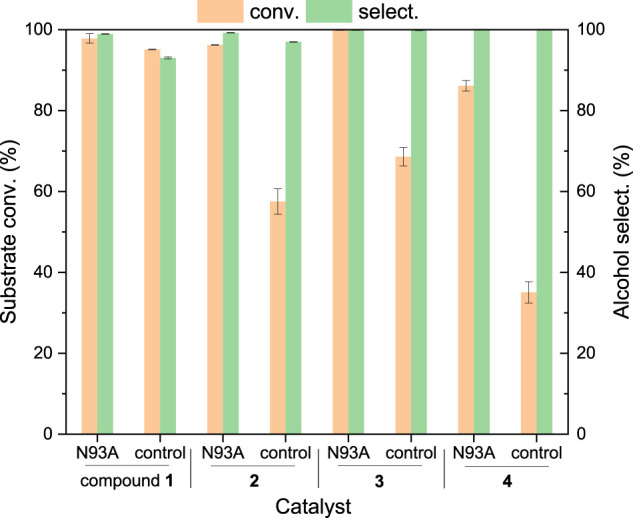
Whole-cell catalytic reduction of biobased furans to furan alcohols. Reaction conditions: 25 mM compounds **1**–**4**, 30 mM glucose, 50 mg/ml (wet weight) recombinant *E. coli* cells, 2 ml Tris–HCl (100 mM, pH 8), 150 rpm, 30°C, 6 h (18 h for HMF transformation); the cells harboring the plain vector worked as controls. The values were obtained from three independent experiments.

To demonstrate the applicability of the engineered enzyme, biocatalytic reduction of biobased furans **1**–**4** to the target alcohols by whole cells harboring variant N93A was performed (Figure 4), with the cells harboring the plain vector as controls. It was found that HMF **1** could be reduced to BHMF in 18 h in the presence of whole cells harboring variant N93A, with the conversion of 98% and selectivity of 99%. A slightly lower conversion (95%) and a slightly lower selectivity (93%) were obtained in control. It suggests the presence of inherent ADHs in *E. coli* cells that enable the HMF reduction. Similarly, the substrate transformation occurred with other biobased furans as substrates in controls. However, the substrate conversions (86–>99%) were higher in the presence of whole cells harboring variant N93A compared to those (35–69%) in controls harboring the plain vector. For example, whole cells harboring variant N93A provide a conversion up to 96% within 6 h in the reduction of furfural **2**, while the conversion of 58% was obtained in control. Higher conversions might be attributed to the introduction of variant N93A to *E. coli* cells. Whole cells harboring variant N93A exhibited significantly different catalytic efficiencies toward furans **1**–**4**, although the activities of variant N93A toward these substrates were comparable (70–83 mU/mg, Table 4). For example, the reaction rate of furfural **2** was much higher than that of HMF **1** (times required for almost complete substrate conversion: 18 vs 6 h), which is in good agreement with our recent results (Cheng et al., 2020). In addition to the activities of variant N93A, the transport rates of substrates across hydrophobic cell membranes might influence the reaction rates in whole-cell catalysis as well (Almeida et al., 2007; Wierckx et al., 2011). More hydrophobic furfural **1** (log *p* value, 0.75) might be more readily transported into the cells for subsequent biotransformation than HMF (−0.10), which may make partial contribution to its higher reaction rate.

## Conclusion

In summary, promiscuous ADH activity of RedAm was considerably improved by replacing only one single amino acid (variant N93A) in this work. *Asp*RedAm showed the NADPH preference in reductive amination, while it favored NADH in promiscuous reduction reactions. Four potential amino acid residues N93, I118, M119, and D169 of *Asp*RedAm were identified by molecular docking for mutagenesis. The thermostability of enzyme was significantly enhanced upon mutation of N93 to alanine. The activities of variant N93A were greatly inhibited in the presence of metal ions tested. The catalytic efficiency of variant N93A was approximately 2-fold higher compared to that of the WT enzyme. The mutant displayed broad substrate specificity. Improved results were obtained with the cells harboring variant N93A in the reduction of biobased furans compared to those in the controls. Nevertheless, the ADH activity of variant N93A is still much lower compared to those of natural ADHs; thus, further molecular modification of this enzyme is still needed via direct evolution and protein engineering for its practical applications in organic synthesis.

## Data Availability

The original contributions presented in the study are included in the article/Supplementary Material; further inquiries can be directed to the corresponding author.

## References

[B1] Aleku G. A. France S. P. Man H. Mangas-Sanchez J. Montgomery S. L. Sharma M. (2017). A reductive aminase from *Aspergillus oryzae* . Nat. Chem. 9 (10), 961–969. 10.1038/nchem.2782 28937665

[B2] Almeida J. R. Röder A. Modig T. Laadan B. Lidén G. Gorwa-Grauslund M. F. (2008). NADH- vs NADPH-coupled reduction of 5-hydroxymethyl furfural (HMF) and its implications on product distribution in *Saccharomyces cerevisiae* . Appl. Microbiol. Biotechnol. 78 (6), 939–945. 10.1007/s00253-008-1364-y 18330568

[B3] Almeida J. R. M. Modig T. Petersson A. Hähn-Hägerdal B. Lidén G. Gorwa-Grauslund M. F. (2007). Increased tolerance and conversion of inhibitors in lignocellulosic hydrolysates by *Saccharomyces cerevisiae* . J. Chem. Technol. Biotechnol. 82 (4), 340–349. 10.1002/jctb.1676

[B4] Amarasekara A. S. Gutierrez Reyes C. D. Obregon R. G. (2020). Biocatalytic reduction of 5-hydroxymethylfurfural to 2,5-furandimethanol using coconut (*Cocos nucifera* L.) water. Biocatal. Agric. Biotechnol. 24, 101551. 10.1016/j.bcab.2020.101551

[B5] Borlinghaus N. Nestl B. M. (2018). Switching the cofactor specificity of an imine reductase. ChemCatChem. 10 (1), 183–187. 10.1002/cctc.201701194

[B6] Bornscheuer U. T. Kazlauskas R. J. (2004). Catalytic promiscuity in biocatalysis: using old enzymes to form new bonds and follow new pathways. Angew. Chem. Int. Ed. Engl. 43 (45), 6032–6040. 10.1002/anie.200460416 15523680

[B7] Bradford M. M. (1976). A rapid and sensitive method for the quantitation of microgram quantities of protein utilizing the principle of protein-dye binding. Anal. Biochem. 72 (1–2), 248–254. 10.1016/0003-2697(76)90527-3 942051

[B8] Brandenberg O. F. Fasan R. Arnold F. H. (2017). Exploiting and engineering hemoproteins for abiological carbene and nitrene transfer reactions. Curr. Opin. Biotechnol. 47, 102–111. 10.1016/j.copbio.2017.06.005 28711855 PMC5617781

[B9] Cheng A.-D. Shi S.-S. Li Y. Zong M.-H. Li N. (2020). Biocatalytic oxidation of biobased furan aldehydes: comparison of toxicity and inhibition of furans toward a whole-cell biocatalyst. ACS Sustain. Chem. Eng. 8 (3), 1437–1444. 10.1021/acssuschemeng.9b05621

[B10] Ducrot L. Bennett M. Grogan G. Vergne-Vaxelaire C. (2020). NAD(P)H-dependent enzymes for reductive amination: active site description and carbonyl-containing compound spectrum. Adv. Synth. Catal. 363, 328–351. 10.1002/adsc.202000870

[B11] Galkin K. I. Ananikov V. P. (2019). When will 5-hydroxymethylfurfural, the “sleeping giant” of sustainable chemistry, awaken? ChemSusChem. 12 (13), 2976–2982. 10.1002/cssc.201900592 31115171

[B12] He Y. C. Jiang C. X. Chong G. G. Di J. H. Ma C. L. (2018). Biological synthesis of 2,5-bis(hydroxymethyl)furan from biomass-derived 5-hydroxymethylfurfural by *E. Coli* CCZU-K14 whole cells. Bioresour. Technol. 247, 1215–1220. 10.1016/j.biortech.2017.09.071 28943097

[B13] Hu L. Xu J. Zhou S. He A. Tang X. Lin L. (2018). Catalytic advances in the production and application of biomass-derived 2,5-dihydroxymethylfuran. ACS Catal. 8, 2959–2980. 10.1021/acscatal.7b03530

[B14] Jia H.-Y. Zong M.-H. Zheng G.-W. Li N. (2019a). Myoglobin-catalyzed efficient in situ regeneration of NAD(P)+ and their synthetic biomimetic for dehydrogenase-mediated oxidations. ACS Catal. 9, 2196–2202. 10.1021/acscatal.8b04890

[B15] Jia H.-Y. Zong M.-H. Zheng G.-W. Li N. (2019b). One-pot enzyme cascade for controlled synthesis of furan carboxylic acids from 5-hydroxymethylfurfural by H_2_O_2_ internal recycling. ChemSusChem. 12 (21), 4764–4768. 10.1002/cssc.201902199 31490638

[B16] Kazlauskas R. J. (2005). Enhancing catalytic promiscuity for biocatalysis. Curr. Opin. Chem. Biol. 9 (2), 195–201. 10.1016/j.cbpa.2005.02.008 15811805

[B17] Khersonsky O. Roodveldt C. Tawfik D. S. (2006). Enzyme promiscuity: evolutionary and mechanistic aspects. Curr. Opin. Chem. Biol. 10 (5), 498–508. 10.1016/j.cbpa.2006.08.011 16939713

[B18] Laadan B. Almeida J. R. Rådström P. Hahn-Hägerdal B. Gorwa-Grauslund M. (2008). Identification of an NADH-dependent 5-hydroxymethylfurfural-reducing alcohol dehydrogenase in *Saccharomyces cerevisiae* . Yeast 25 (3), 191–198. 10.1002/yea.1578 18302314

[B19] Lenz M. Fademrecht S. Sharma M. Pleiss J. Grogan G. Nestl B. M. (2018). New imine-reducing enzymes from β-hydroxyacid dehydrogenases by single amino acid substitutions. Protein Eng. Des. Sel. 31 (4), 109–120. 10.1093/protein/gzy006 29733377

[B20] Lenz M. Meisner J. Quertinmont L. Lutz S. Kästner J. Nestl B. M. (2017). Asymmetric ketone reduction by imine reductases. ChemBioChem. 18 (3), 253–256. 10.1002/cbic.201600647 27911981

[B21] Leveson-Gower R. B. Mayer C. Roelfes G. (2019). The importance of catalytic promiscuity for enzyme design and evolution. Nat. Rev. Chem. 3 (12), 687–705. 10.1038/s41570-019-0143-x

[B22] Li X. Jia P. Wang T. (2016). Furfural: a promising platform compound for sustainable production of C4 and C5 chemicals. ACS Catal. 6 (11), 7621–7640. 10.1021/acscatal.6b01838

[B23] Li Y. M. Zhang X. Y. Li N. Xu P. Lou W. Y. Zong M. H. (2017). Biocatalytic reduction of HMF to 2,5-bis(hydroxymethyl)furan by HMF-tolerant whole cells. ChemSusChem. 10 (2), 372–378. 10.1002/cssc.201601426 27966286

[B24] Liu L. Wang D.-H. Chen F.-F. Zhang Z.-J. Chen Q. Xu J.-H. (2020). Development of an engineered thermostable amine dehydrogenase for the synthesis of structurally diverse chiral amines. Catal. Sci. Technol. 10 (8), 2353–2358. 10.1039/D0CY00071J

[B25] Mariscal R. Maireles-Torres P. Ojeda M. Sádaba I. López Granados M. (2016). Furfural: a renewable and versatile platform molecule for the synthesis of chemicals and fuels. Energy Environ. Sci. 9 (4), 1144–1189. 10.1039/C5EE02666K

[B26] Petri A. Masia G. Piccolo O. (2018). Biocatalytic conversion of 5-hydroxymethylfurfural: synthesis of 2,5-bis(hydroxymethyl)furan and 5-(hydroxymethyl)furfurylamine. Catal. Commun. 114, 15–18. 10.1016/j.catcom.2018.05.011

[B27] Qin Y.-Z. Li Y.-M. Zong M.-H. Wu H. Li N. (2015). Enzyme-catalyzed selective oxidation of 5-hydroxymethylfurfural (HMF) and separation of HMF and 2,5-diformylfuran using deep eutectic solvents. Green Chem. 17 (7), 3718–3722. 10.1039/C5GC00788G

[B28] Roth S. Präg A. Wechsler C. Marolt M. Ferlaino S. Lüdeke S. (2017). Extended catalytic scope of a well-known enzyme: asymmetric reduction of iminium substrates by glucose dehydrogenase. ChemBioChem. 18 (17), 1703–1706. 10.1002/cbic.201700261 28722796

[B29] Sharma M. Mangas-Sanchez J. Turner N. J. Grogan G. (2017). NAD(P)H-dependent dehydrogenases for the asymmetric reductive amination of ketones: structure, mechanism, evolution and application. Adv. Synth. Catal. 359 (12), 2011–2025. 10.1002/adsc.201700356 30008635 PMC6033044

[B30] Sheldon R. A. (2018). Chemicals from renewable biomass: a renaissance in carbohydrate chemistry. Curr. Opin. Green Sustain. Chem. 14, 89–95. 10.1016/j.cogsc.2018.08.003

[B31] Sheldon R. A. (2014). Green and sustainable manufacture of chemicals from biomass: state of the art. Green Chem. 16 (3), 950–963. 10.1039/C3GC41935E

[B32] Shen G. Andrioletti B. Queneau Y. (2020). Furfural and 5-(hydroxymethyl)furfural: two pivotal intermediates for bio-based chemistry. Curr. Opin. Green Sustain. Chem. 26, 100384. 10.1016/j.cogsc.2020.100384

[B33] Svedendahl M. Hult K. Berglund P. (2005). Fast carbon-carbon bond formation by a promiscuous lipase. J. Am. Chem. Soc. 127 (51), 17988–17989. 10.1021/ja056660r 16366534

[B34] Tseliou V. Knaus T. Masman M. F. Corrado M. L. Mutti F. G. (2019). Generation of amine dehydrogenases with increased catalytic performance and substrate scope from ε-deaminating L-Lysine dehydrogenase. Nat. Commun. 10 (1), 3717. 10.1038/s41467-019-11509-x 31420547 PMC6697735

[B35] Tseliou V. Schilder D. Masman M. F. Knaus T. Mutti F. (2020). Generation of oxidoreductases with dual alcohol dehydrogenase and amine dehydrogenase activity. Chemistry 27, 3315–3325. 10.1002/chem.202003140 33073866 PMC7898336

[B36] Tuck C. O. Pérez E. Horváth I. T. Sheldon R. A. Poliakoff M. (2012). Valorization of biomass: deriving more value from waste. Science 337 (6095), 695–699. 10.1126/science.1218930 22879509

[B37] van Putten R. J. van der Waal J. C. de Jong E. Rasrendra C. B. Heeres H. J. de Vries J. G. (2013). Hydroxymethylfurfural, a versatile platform chemical made from renewable resources. Chem. Rev. 113 (3), 1499–1597. 10.1021/cr300182k 23394139

[B38] Wang X. Zhang X.-Y. Zong M.-H. Li N. (2020). Sacrificial substrate-free whole-cell biocatalysis for the synthesis of 2,5-furandicarboxylic acid by engineered *Escherichia coli* . ACS Sustain. Chem. Eng. 8, 4341–4345. 10.1021/acssuschemeng.0c00058

[B39] Wen M. Zhang X.-Y. Zong M.-H. Li N. (2020). Significantly improved oxidation of bio-based furans into furan carboxylic acids using substrate-adapted whole cells. J. Energy Chem. 41, 20–26. 10.1016/j.jechem.2019.04.025

[B40] Wierckx N. Koopman F. Ruijssenaars H. J. de Winde J. H. (2011). Microbial degradation of furanic compounds: biochemistry, genetics, and impact. Appl. Microbiol. Biotechnol. 92 (6), 1095–1105. 10.1007/s00253-011-3632-5 22031465 PMC3223595

[B41] Yan Y. Bu C. Huang X. Ouyang J. (2019). Efficient whole‐cell biotransformation of furfural to furfuryl alcohol by *Saccharomyces cerevisiae* NL22. J. Chem. Technol. Biotechnol. 94 (12), 3825–3831. 10.1002/jctb.6177

[B42] Zhang X.-Y. Xu Z.-H. Zong M.-H. Wang C.-F. Li N. (2019). Selective synthesis of furfuryl alcohol from biomass-derived furfural using immobilized yeast cells. Catalysts 9 (1), 70. 10.3390/catal9010070

[B43] Zhang X.-Y. Zong M.-H. Li N. (2017). Whole-cell biocatalytic selective oxidation of 5-hydroxymethylfurfural to 5-hydroxymethyl-2-furancarboxylic acid. Green Chem. 19, 4544–4551. 10.1039/C7GC01751K

[B44] Zhang X. Y. Ou X. Y. Fu Y. J. Zong M. H. Li N. (2020a). Efficient synthesis of 5-hydroxymethyl-2-furancarboxylic acid by *Escherichia coli* overexpressing aldehyde dehydrogenases. J. Biotechnol. 307, 125–130. 10.1016/j.jbiotec.2019.11.007 31726082

[B45] Zhang X. Y. Wang X. Li N. W. Guo Z. W. Zong M. H. Li N. (2020b). Furan carboxylic acids production with high productivity by cofactor‐engineered whole‐cell biocatalysts. ChemCatChem. 12, 3257–3264. 10.1002/cctc.202000259

