# Supplementary material for: Engineering Promiscuous Alcohol Dehydrogenase Activity of a Reductive Aminase AspRedAm for Selective Reduction of Biobased Furans
Source: Front Chem. 2021 May 13;9:610091. doi: 10.3389/fchem.2021.610091 (PMC8155666; doi:10.3389/fchem.2021.610091)
Supplement: Supplementary file 1 [file DataSheet1.PDF]

Supplementary Material

1 Cloning and Heterologous Expression of Enzyme

The codon-optimized *Asp*RedAm gene (GenBank accession number KY327363)(Aleku et al., 2017) was synthesized by Sangon Biotech Co., Ltd. (Shanghai, China), which was inserted into the plasmid pUC57m (pUC57m-*Asp*RedAm). The plasmids pUC57m-AspRedAm and pET-28a were double-digested using the restriction enzymes *Nde*I and *Xho*I; then, the resulting products were ligated by T4 DNA ligase, thus generating the recombinant plasmid pET-28a-*Asp*RedAm. The plasmids pET-28a-*Asp*RedAm and pET-28a-variant were transformed into *E. coli* BL21(DE3), and pre-cultivated in 20 mL LB medium (5 g/L yeast extract, 10 g/L tryptone, and 10 g/L NaCl) containing 30 mg/L kanamycin at 37°C and 180 rpm overnight. Then, 100 mL LB medium containing 30 mg/L kanamycin was inoculated with 1 mL of an overnight culture. Cells were grown at 37 °C and 180 rpm. When the optical density at 600 nm (OD_{600}) reached 0.6-0.8, IPTG was added for inducing protein expression at the final concentration of 0.2 mM, followed by incubation at 20 °C for 20 h and 160 rpm. The cells were harvested by centrifugation (8000 r/min, 5 min, 4 °C) and washed twice with 0.85% NaCl solution.

2 Site-Directed Mutagenesis

Whole plasmid PCR was used to introduce mutations to the *Asp*RedAm gene by site-directed mutagenesis. The primers (Supplementary Table 1) used for mutation were synthesized by Majorbio Inc. (Shanghai, China). PCR was mediated by Q5 High-Fidelity DNA polymerase in the presence of both forward and reverse primers, with pET-28a-*Asp*RedAm as the template. The PCR products were subjected to *Dpn*I digestion to remove the template DNA. The resulting products were transformed to *E. coli* DH5α, followed by screening the positive transformants. The plasmids was extracted from the positive transformants for sequencing by Majorbio Inc. (Shanghai, China). The verified plasmids were transformed to *E. coli* BL21(DE3) for heterologously expressing variants.

Supplementary Table 1. Primers used for mutation

Mutant		Primer sequence (5’-3’)
N93A	F	ACCTGACTGCAGGTACTCCGAACCAGGCGC
	R	AGTACCTGCAGTCAGGTTAACGATGGTTTTAGACG
N93S	F	ACCTGACTAGCGGTACTCCGAACCAGGCGCGT
	R	AGTACCGCTAGTCAGGTTAACGATGGTTTTAGACG
N93Q	F	ACCTGACTCAGGGTACTCCGAACCAGGCGCGT
	R	AGTACCGTCAGTCAGGTTAACGATGGTTTTAGACG
I118N	F	TCCACGGTGGTAATATGGCGGTGCCGACCATG
	R	CCATATTACCACCGTGGATGTAACGTGC
M119S	F	ATCCACGGTGGTATCAGCGCGGTGCCGACCATGATT
	R	GCTGATACCACCGTGGATGTAACG
D169A	F	ATGCACTGGCACTGCTGTCTGGCA
	R	ACAGCAGTGCCAGTGCATGCAGGCTCGCAGAGC
D169S	F	CTGCATAGCCTGGCACTGCTGTCTGGCA
	R	AGTGCCAGGCTATGCAGGCTCGCAGAGCC
D169K	F	CCTGCATAAACTGGCACTGCTGTCTGGCA
	R	GTGCCAGTTTATGCAGGCTCGCAGAGCC

3 Enzyme Purification

Binding Buffer: 100 mM Tris-HCl, 500 mM NaCl, 50 mM imidazole, pH 8.
Elution Buffer: 100 mM Tris-HCl, 500 mM NaCl, 500 mM imidazole, pH 8.
Ni-affinity chromatography: The harvested cells were re-suspended in the Binding Buffer in the cell concentration of 0.1 g/mL (wet weight), followed by ultrasonication (350 w; 3×5 min, 2 second ON, 3 second OFF cycles). Upon centrifugation, the supernatant was loaded onto a HisTrap HP column (GE Healthcare, USA) that was pre-equilibrated with the Binding Buffer according to the manufacturer´s instructions. The His_6-tagged protein was purified by Ni-affinity chromatography using a Bio-Rad NGC QuestTM10 purifier, by gradually increasing the Elution Buffer contents. the obtained protein was desalted using a HiTrap Desalting column (GE Healthcare, USA), with Tris-HCl buffer (100 mM, pH 8) as the eluent.

Supplementary Table 2. The ADH activities of *Asp*RedAm and its variants

	*Asp*RedAm	N93A	N93S
ADH activity (mU/mg)	0.8 ± 0.0	5.3 ± 0.3	0.8 ± 0.0

Reaction conditions: 5 mM HMF, 0.2 mM NADPH, appropriate amount of enzyme, 0.4 mL Tris-HCl (100 mM, pH 9), 30 °C.

Supplementary Table 3. Effect of cofactors on the RedAm activities of *Asp*RedAm

Amine	Enzyme activity (mU/mg)			
	HMF		Cyclohexanone	
	NADH	NADPH	NADH	NADPH
Propargylamine	5.3 ± 0.5	12.4 ± 1.2	74 ± 8	6360 ± 36
Methylamine	2.5 ± 0.3	9.3 ± 1.8	24 ± 3	2680 ± 39

Reaction conditions: 15 mM substrate, 60 mM amine donor, 0.3 mM NAD(P)H, 43 μg/mL *Asp*RedAm, 0.4 mL borate buffer (100 mM, pH 8), 30 ℃; final pH of the reactant mixture was tuned to 9 before addition of enzyme.

4 Molecular docking

Molecular docking was performed to identify the key residues for semi-rational engineering. The substrate HMF was docked to the crystal structure of wide type *Asp*RedAm (PDB ID: 5G6R) using Autodock 4.2, generating 250 docking poses. Configuration clusters were analyzed for the reasonable binding mode with the highest docking energy. Pymol was then used to visualize the *Asp*RedAm structure in complex with HMF, and residues within 4 Å around the substrate HMF were selected as key residues for further experimental verification.

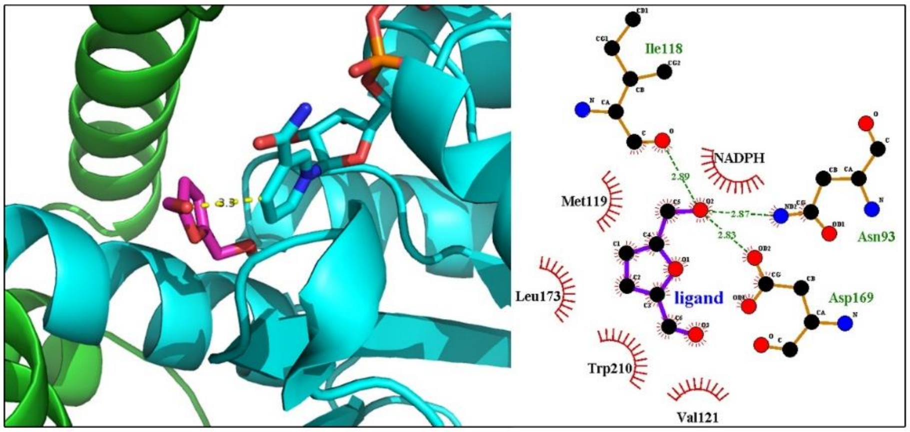

Supplementary Figure 1. The results of *Asp*RedAm docking with HMF and cofactor

5 HPLC Chromatograms

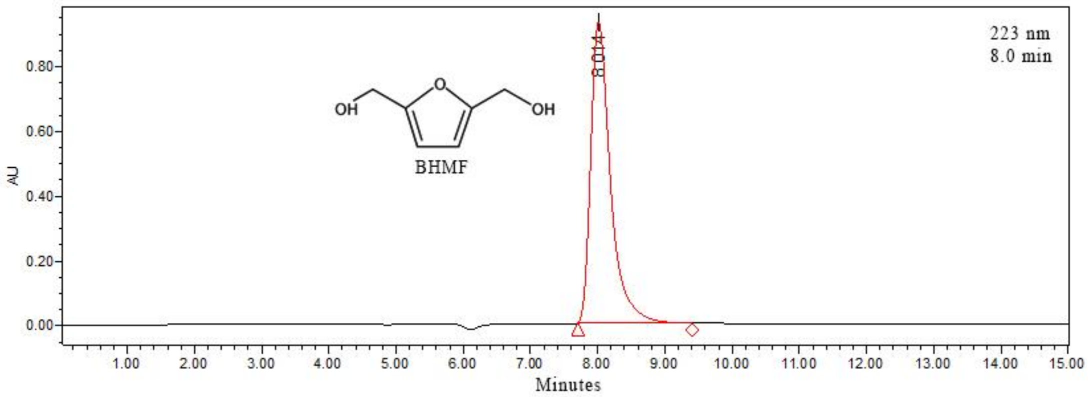
223 nm
8.0 min
8.014
BHMF
OH
OH
AU
Minutes

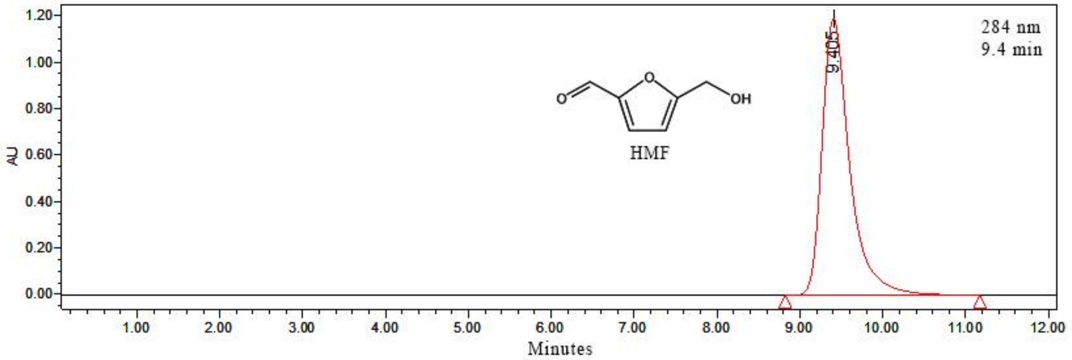
284 nm
9.4 min
9.405
HMF
OH
AU
Minutes

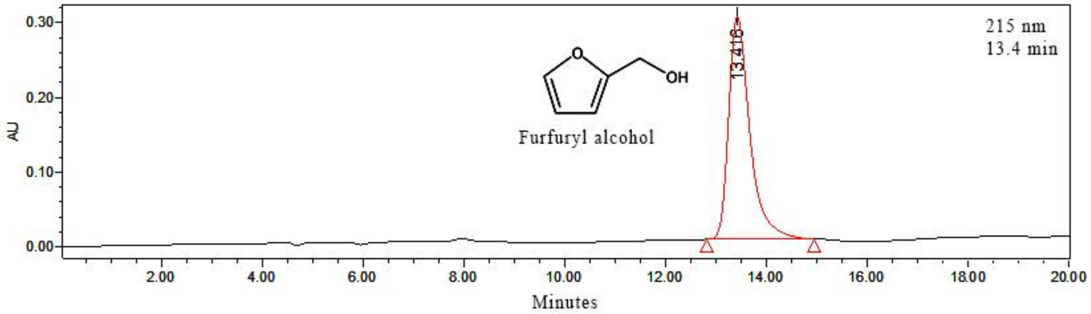
215 nm
13.4 min
13.418
Furfuryl alcohol
OH
AU
Minutes

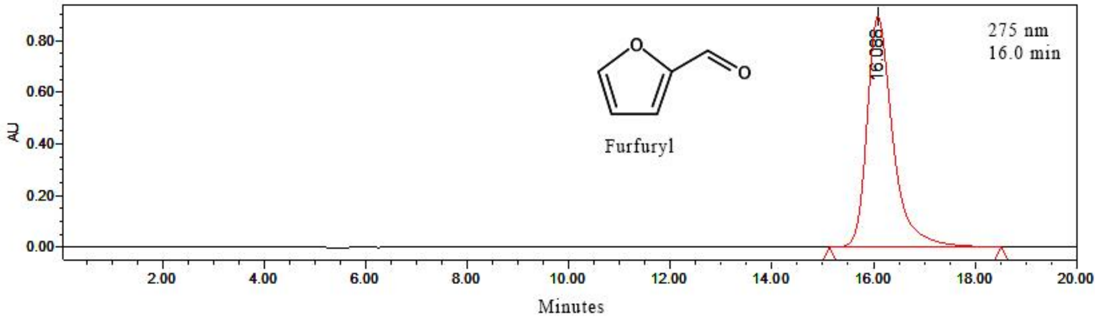
275 nm
16.0 min
16.088
Furfuryl
AU
Minutes

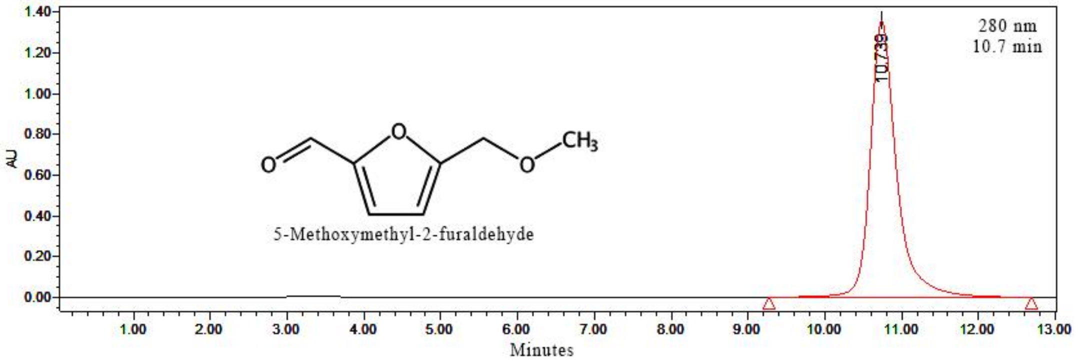

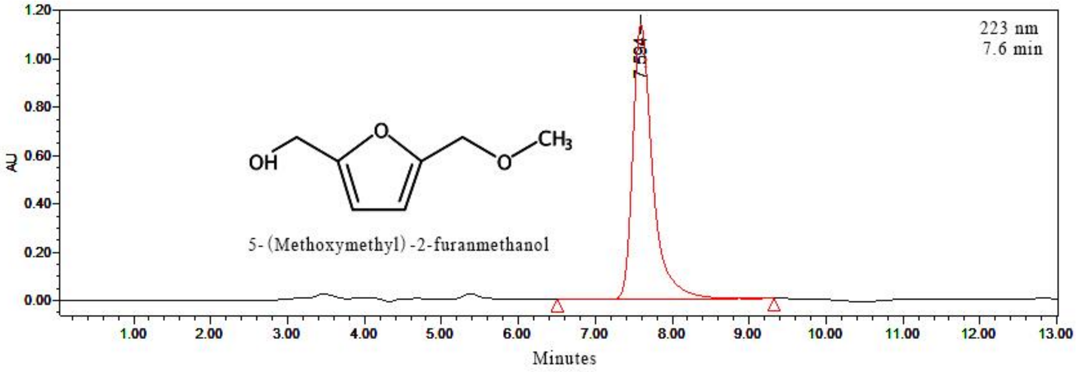

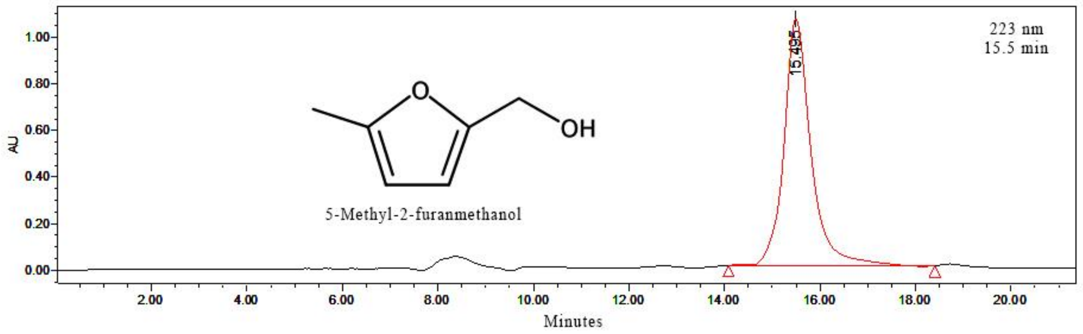

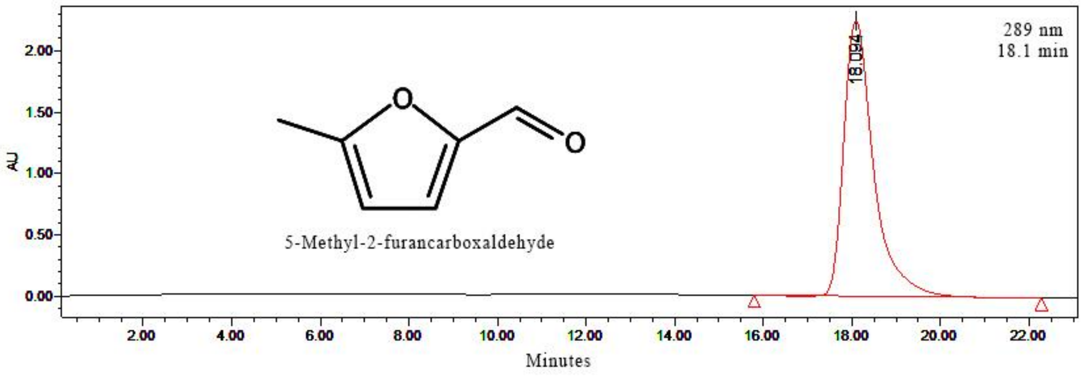

6 Calibration curves of representative substrates and products

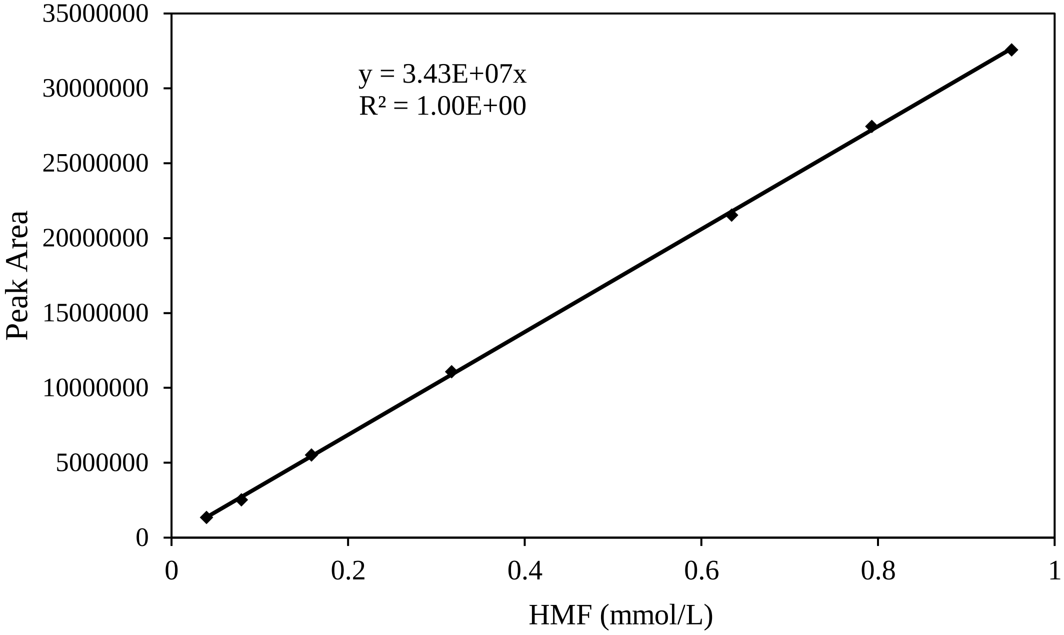

HMF calibration curve

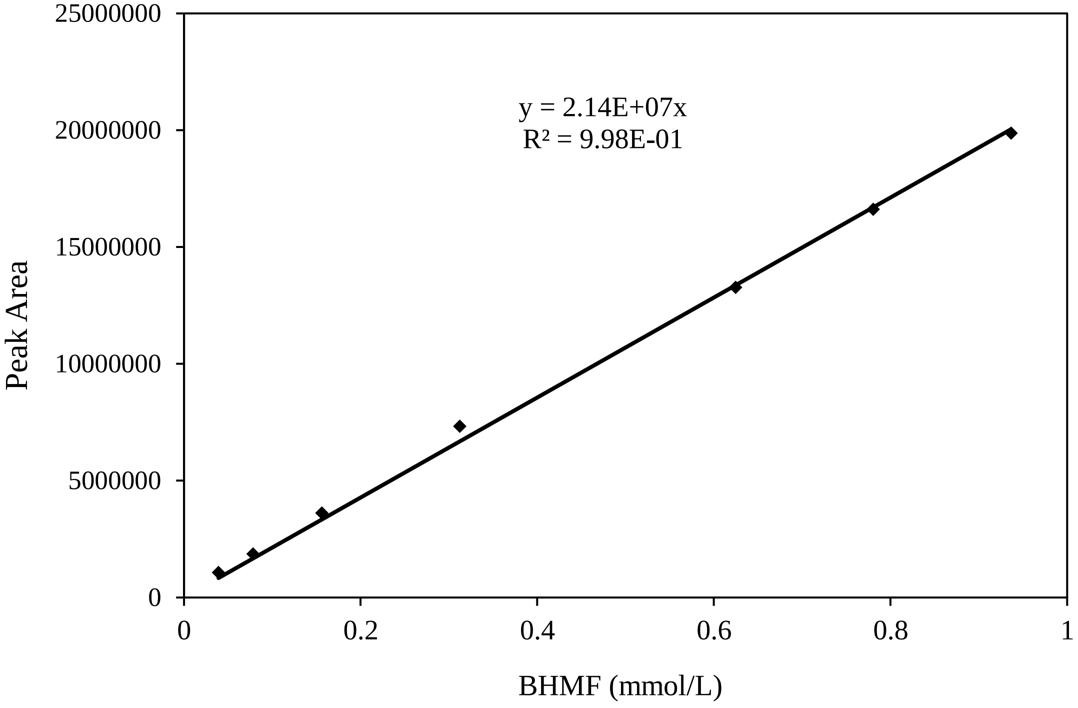

BHMF calibration curve

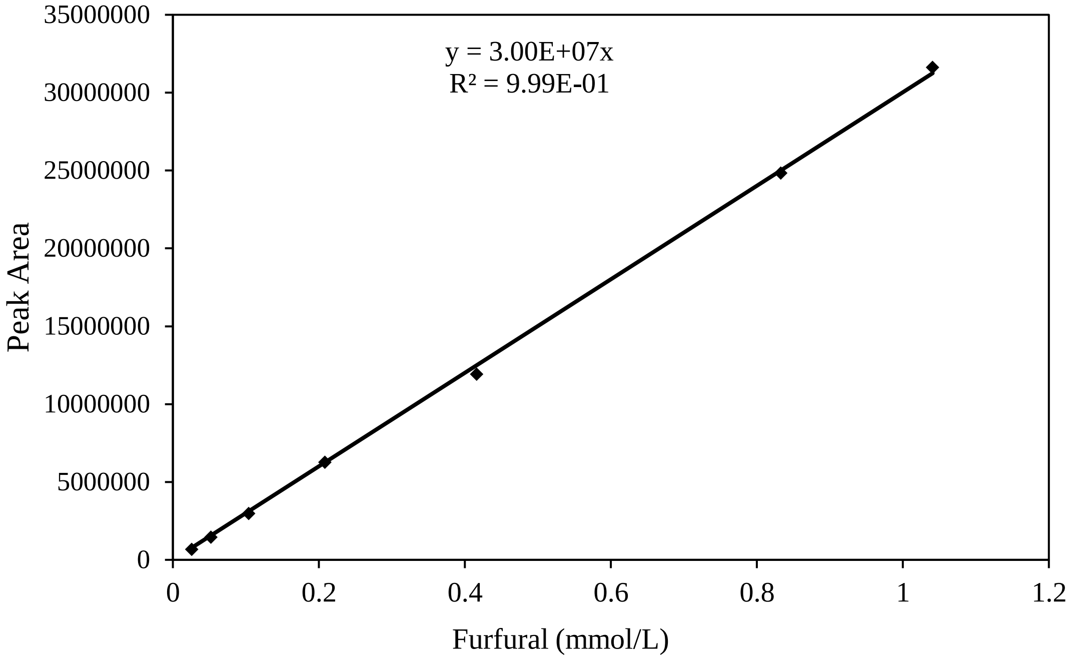

Furfural calibration curve

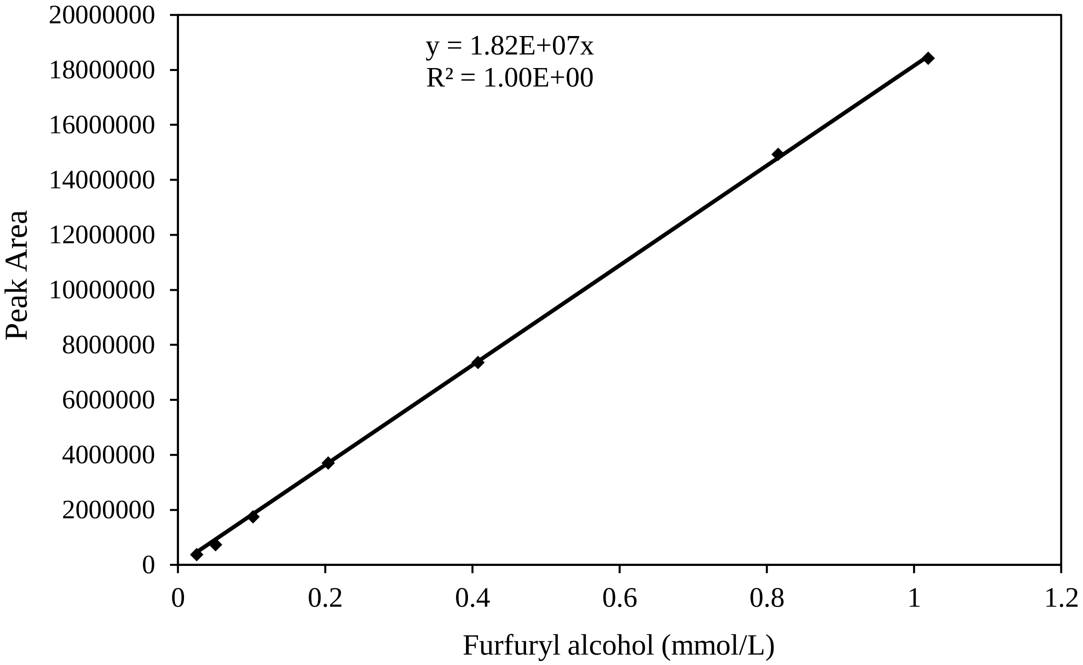

Furfuryl alcohol calibration curve

Reference

Aleku, G.A., France, S.P., Man, H., Mangas-Sanchez, J., Montgomery, S.L., Sharma, M., et al. (2017). A reductive aminase from *aspergillus oryzae*. *Nat. Chem.* 9(10)**,** 961-969. doi: 10.1038/nchem.2782.
